# Volar locking and angular stability plate for treatment of comminuted scaphoid fractures: a case series of 44 cases

**DOI:** 10.1007/s00590-024-04095-2

**Published:** 2024-09-20

**Authors:** L. Marzella, S. Filistad, A. Lazzerini, A. Cannella, G. Sassara, L. Caruso, R. De Vitis

**Affiliations:** 1 Unit of Hand Surgery, IRCCS Istituto Ortopedico Galeazzi, Milan, Italy; 2 Department of Orthopaedics, Fondazione Policlinico Universitario A. Gemelli, Largo A. Gemelli 8, 00168 Rome, Italy

**Keywords:** Scaphoid fracture, Volar locking plate, Angular stability, Scaphoid plating, Comminuted fracture

## Abstract

**Background:**

The primary method employed worldwide for the treatment of scaphoid fractures is screw fixation. However, in unstable and comminuted fractures, percutaneous fixation could produce complications due to technical challenges, such as improper axis positioning, inaccurate screw length measurement, intra-articular screw penetration, and impingement. Alternative open approaches for the surgical management of scaphoid fractures have been proposed, and in recent years, a new specific volar locking plate for the treatment of scaphoid fractures has been developed. This study aims to present the outcomes of this technique applied to 44 patients with unstable and comminuted fractures of the scaphoid.

**Aims:**

The purpose of the study is to verify the effectiveness of the volar plate in the treatment of comminuted scaphoid fractures and the necessity for plate removal when consolidation has occurred.

**Methods:**

Between January 2021 and March 2023, a specific volar locking plate for the treatment of scaphoid fractures was used in 44 patients. A retrospective study was conducted involving all patients, consisting of continuous clinical and radiographic assessments, functional evaluations (using QuickDASH and MHQ), and patient satisfaction surveys.

**Results:**

All patients achieved clinical and radiographic recovery. However, the plate can impinge with nearby structures and should be removed once the fracture is consolidated. After plate removal, further improvement in range of motion was observed.

**Conclusion:**

The plate and screws system is a viable and appropriate method of osteosynthesis in the treatment of unstable and comminuted recent fractures occurring in the middle third of the carpal scaphoid.

## Introduction

Scaphoid fractures are common and challenging hand injuries due to the typical anatomical characteristics of the scaphoid. Treatment must consider the particular bone vascularization of the scaphoid, with approximately 80% of its surface covered by articular cartilage, and its irregular and variable shape. Different classifications of scaphoid fractures are available in the literature, with Herbert’s classification being the most commonly used. This classification describes two categories (A and B) based on the location and orientation of the fracture rim [1]. Stable fractures (type A) can typically be managed conservatively, while unstable fractures (type B) require surgical intervention due to historically high failure rates associated with conservative treatment.

For unstable fractures (type B), surgical intervention has become the standard of care in contemporary practice. This approach is essential due to the historically documented high failure rates associated with conservative treatment, as reported in the medical literature. In some cases, these failure rates are as high as 92% in unstable fractures with an initial displacement exceeding one millimeter [2], which is why surgical treatment became the gold standard.

Surgical management plays a critical role in achieving a reliable union, preventing avascular necrosis of the proximal pole, and averting rapid progression to pan-carpal arthritis. [3].

The primary method employed worldwide for the treatment of scaphoid fractures is screw fixation. [4, 5]. However, in unstable and comminuted fractures, screw fixation may lead to complications such as improper axis positioning, inaccurate screw length measurement, intra-articular screw penetration, and impingement [6]. Alternative open approaches for surgical management, including the use of staples, have been proposed, but staples have not gained widespread acceptance among orthopedic and hand surgeons for scaphoid treatment. In recent years, a new specific volar locking and angular stability plate for the treatment of scaphoid fractures has been developed [7–10].

In the last years was produced a new specific volar locking and angular stability plate for treatment of scaphoid fractures.

The authors present results and consideration about a case series of 44 comminuted scaphoid fractures treated with volar locking and angular stability plate.

## Materials and methods

The management of unstable scaphoid fractures has been a longstanding practice at the Divisions of Hand Surgery of both Galeazzi Hospital IRCCS in Milan and Policlinic “A. Gemelli” foundation IRCCS in Rome. Initially, Herbert screws were employed for fixation, but since the introduction of new plating systems, open reduction and plate and screw osteosynthesis have been implemented especially for the treatment of those fractures with bone loss and/or multiple fragments.

This transition in methodology of surgical treatment of comminuted and unstable fracture was driven by specific attributes of this fixation technique, such as the capacity for a completely extra-articular implant (while preserving the scapho-trapezial and scapho-radial joint surfaces), reduced bone exposure compared to the original Herbert open focus system, direct visualization of the fracture, and procedural simplicity.

**Fig. 1:**
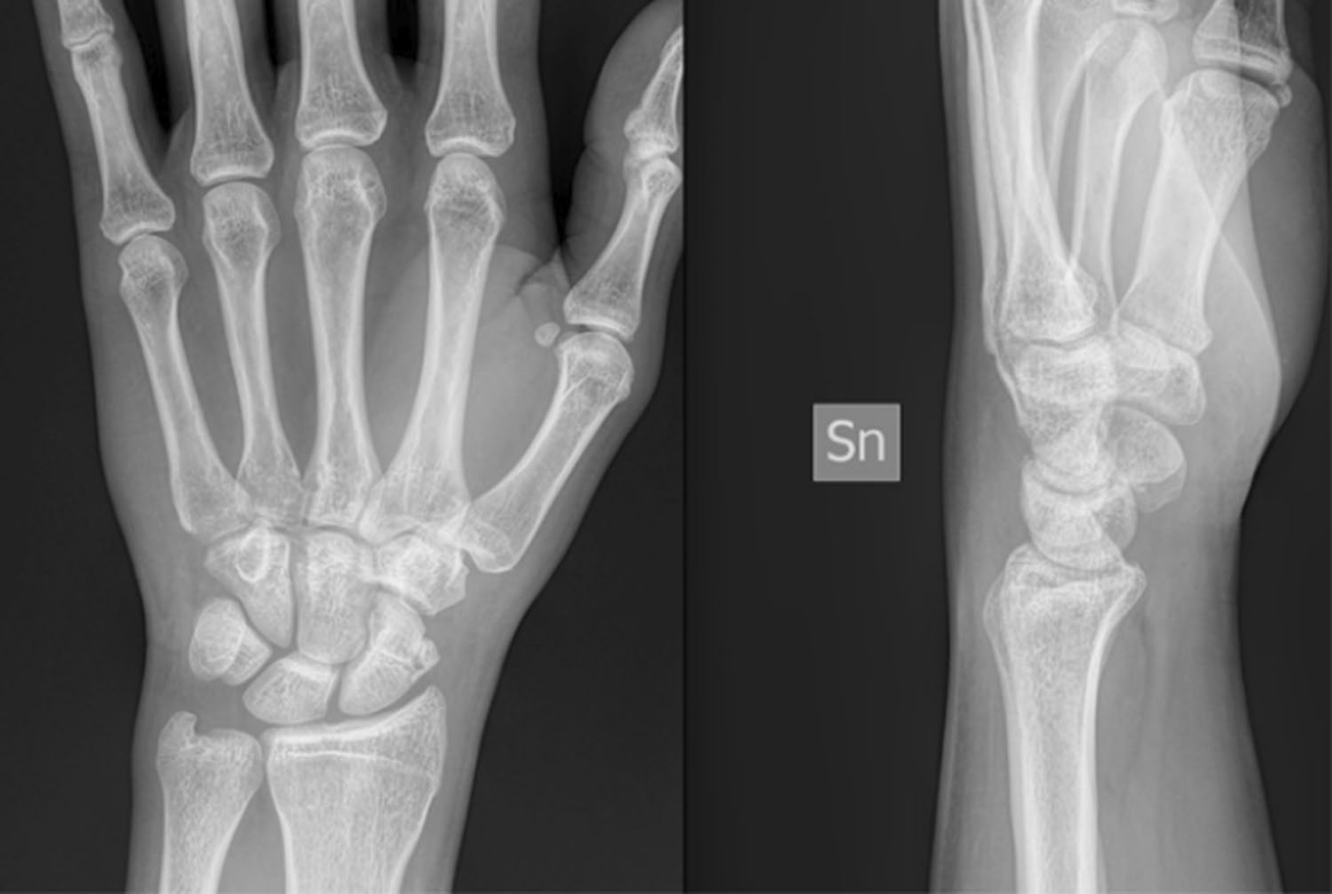
Pre-operative X-rays

**Fig. 2:**
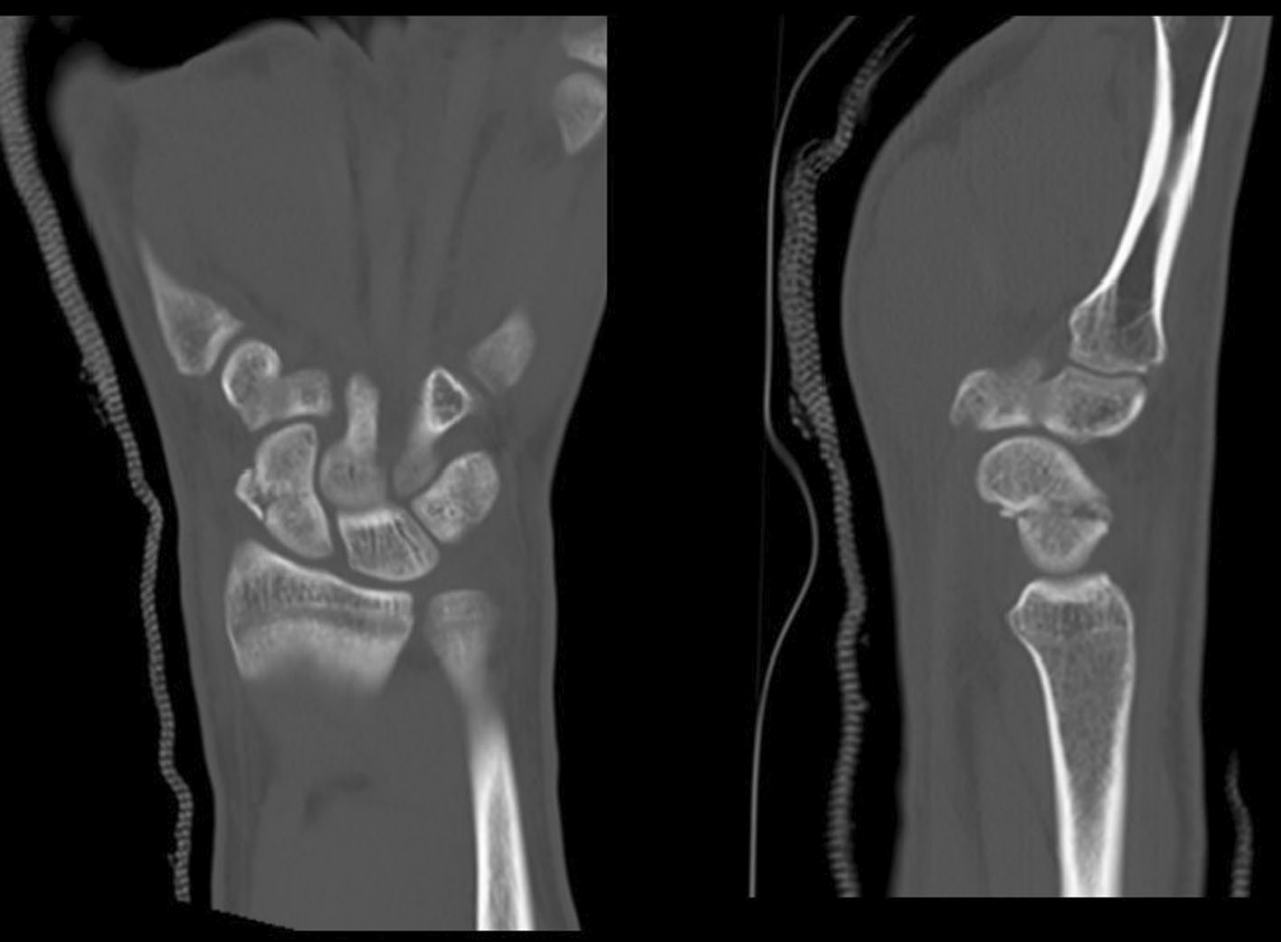
Pre-operative TC

Between January 2021 and March 2023, approximately 500 scaphoid fractures were admitted to the two hospitals of which 44 (8%) presented as comminuted fractures of the scaphoid body and were treated with a pre-contoured volar locking and angular stability plate 1.5 mm (Medartis) specific for the treatment of scaphoid fractures. Plate and screws were constructed from biocompatible nickel-titanium materials. Although there is no satisfactory classification regarding this type of fractures, should be classified like Type B5 of Herbert (Figs. 1 and 2). This retrospective study was performed in respect of the guidelines of both the institutional ethic committees of experimental protocols of Galeazzi Hospital IRCCS and Policlinic “A. Gemelli” foundation IRCCS. All the patients enrolled in this study sing an informed consent at Hospital admission.

**Fig. 3:**
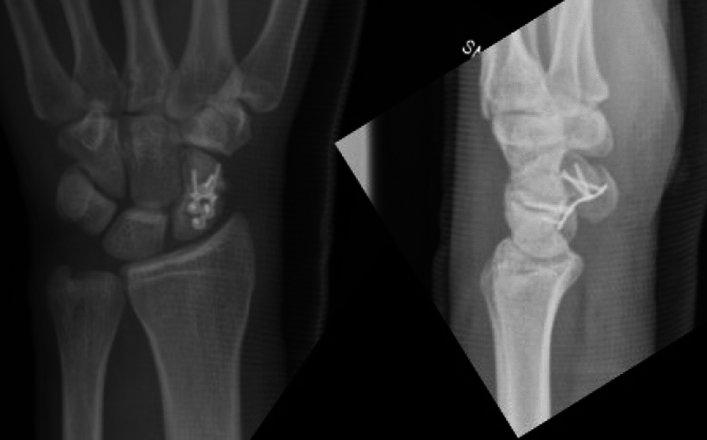
Intraoperative X-rays

**Fig. 4:**
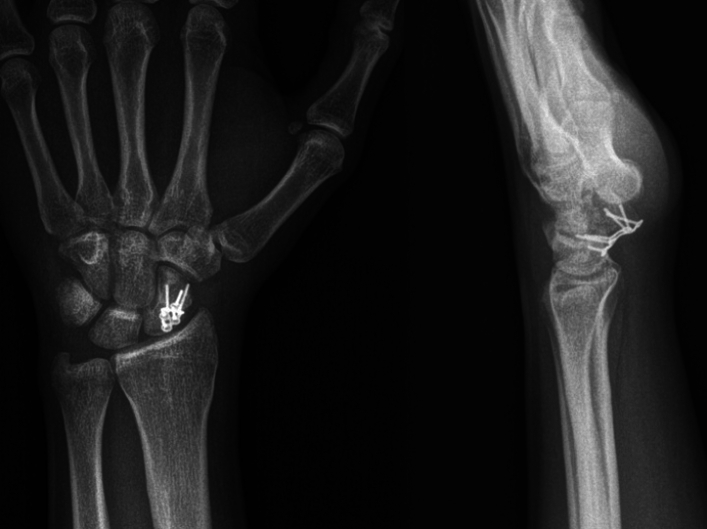
1 year follow-up X-rays

Following surgery, all patients were immobilized with a short forearm cast for the first 3 to 6 postoperative weeks. Subsequently, after suture and cast/ immobilization removal, they were permitted controlled wrist movement until radiographic consolidation was achieved. Radiographic consolidation occurred within 60–90 days in all cases (Figs. 3 and 4).

A retrospective study was conducted involving all 44 patients, which consisted of serialized continuous clinical and radiographic assessments at intervals of 2 weeks, and at 3-, 6- and 12-months after osteosynthesis and 4 months after plate removal. The patient selection process did not involve specific criteria, except for the ability to monitor patients throughout the study duration. No patients were lost to follow-up during the study period (Tables 1, 2 and 3).

**Table 1:** Demographic characteristics of the patients

Patients numerosity	44	
Sex (M–F)	(44–0)	
Age (mean, range)	35.0 (14–56) years old	
Causes of fracture	Car accident	20 (46%)
	Bike accident	12 (28%)
	Goalkeeping	9 (19%)
	Football injury	3 (7%)
Herbert classification	A1	0
	A2	0
	B1	0
	B2	0
	B3	0
	B4	0
	B5	44
	C	0
	D1	0
	D2	0

**Table 2:** ROM before and after plate removal

PZ/ROM	Flexion before plate removal	Flexion 4 months after plate removal	Extension before plate removal	Extension 4 months after plate removal
1	60,00	80,00	80,00	80,00
2	65,00	80,00	80,00	80,00
3	60,00	80,00	70,00	70,00
4	60,00	90,00	70,00	80,00
5	80,00	90,00	80,00	80,00
6	60,00	80,00	70,00	70,00
7	65,00	80,00	70,00	80,00
8	50,00	70,00	70,00	80,00
9	50,00	70,00	65,00	80,00
10	55,00	80,00	80,00	80,00
11	60,00	90,00	65,00	80,00
12	70,00	90,00	80,00	80,00
13	90,00	90,00	80,00	80,00
14	60,00	90,00	80,00	80,00
15	75,00	90,00	80,00	80,00
16	60,00	75,00	60,00	80,00
17	60,00	80,00	70,00	80,00
18	80,00	90,00	80,00	80,00
19	50,00	70,00	60,00	70,00
20	60,00	80,00	60,00	80,00
21	50,00	70,00	70,00	80,00
22	50,00	75,00	80,00	80,00
23	80,00	90,00	80,00	80,00
24	60,00	75,00	50,00	70,00
25	50,00	80,00	80,00	80,00
26	60,00	90,00	70,00	80,00
27	60,00	90,00	70,00	70,00
28	60,00	75,00	80,00	80,00
29	60,00	75,00	80,00	80,00
30	65,00	90,00	80,00	80,00
31	60,00	90,00	80,00	80,00
32	60,00	85,00	70,00	80,00
33	60,00	75,00	80,00	80,00
34	65,00	90,00	80,00	80,00
35	60,00	75,00	80,00	80,00
36	55,00	80,00	80,00	80,00
37	70,00	90,00	80,00	80,00
38	65,00	90,00	80,00	80,00
39	55,00	90,00	80,00	80,00
40	60,00	85,00	70,00	80,00
41	70,00	90,00	70,00	80,00
42	70,00	90,00	70,00	80,00
43	60,00	90,00	80,00	80,00
44	60,00	85,00	75,00	80,00

**Table 3:** QDASH scores before and after plate removal

PZ/QDASH	QDASH before plate removal	QDASH 4 months after plate removal	QDASH net improvement
1	50,00	6,82	43,18
2	68,18	18,18	50,00
3	61,36	22,73	38,63
4	57,50	6,82	50,68
5	63,64	15,91	47,73
6	50,00	15,91	34,09
7	57,5	6,82	50,68
8	52,27	6,82	45,45
9	68,18	9,09	59,09
10	47,73	11,36	36,37
11	57,5	6,82	50,68
12	43,18	18,18	25,00
13	63,64	15,91	47,73
14	50,00	9,09	40,91
15	45,45	9,09	36,36
16	50,00	6,82	43,18
17	45,45	22,73	22,72
18	47,73	6,82	40,91
19	52,5	18,18	34,32
20	63,64	6,82	56,82
21	61,36	6,82	54,54
22	52,27	18,18	34,09
23	25,00	18,18	6,82
24	50,00	11,36	38,64
25	72,73	13,64	59,09
26	40,91	22,73	18,18
27	54,55	6,82	47,73
28	63,64	9,09	54,55
29	72,73	18,18	54,55
30	50,00	6,82	43,18
31	57,5	6,82	50,68
32	68,18	6,82	61,36
33	68,18	22,73	45,45
34	57,5	6,82	50,68
35	61,36	31,82	29,54
36	50,00	15,91	34,09
37	63,64	6,82	56,82
38	63,64	15,91	47,73
39	15,91	13,64	2,27
40	54,60	12,73	41,87
41	25,00	9,09	15,91
42	54,55	18,18	36,37
43	68,18	11,36	56,82
44	57,5	6,82	50,68
Mean values	54,65 ± 12,20	12,69 ± 6,28	41,96 ± 13,82

### Surgical techniques

In the surgical management of scaphoid fractures, a palmar approach through the flexor carpi radialis sheath is employed. This approach includes a longitudinal capsule incision, allowing for direct visualization of the fracture site. Following manual reduction of the fracture; hyperextension of the wrist helps in exposing the scaphoid to better identify the borders. A mild traction of the thumb can help with fracture reduction and plate positioning. K-wires are then used to stabilize the plate in the desired position. As the plate is positioned locking and compression screws are oriented to lock and stabilize every fragment of the fracture. It is advisable to start fixation by positioning the two opposite screws into the poles of the scaphoid, after that the K wires are removed, and reduction is confirmed under fluoroscope. If the plate is in the right position all the other screws are placed. It’s always advisable to underestimate the screw length to avoid tip protrusion into adjacent joints.

The joint capsule and skin are meticulously sutured, and wrist immobilization with a short forearm cast is maintained for a duration from 3 to 6 weeks depending on the stability achieved with the plate. After that controlled wrist movement was allowed until radiographic consolidation was achieved within 60–90 days in all cases.

The plate can impinge with the nearby structures and should be removed once the fracture is consolidated.

### Statistical analysis

The Kolmogorov–Smirnov test was used to determine the normal distribution of da-ta. A paired t test was performed to evaluate the difference between the preoperative and postoperative values. A *P*-value of < 0.05 was considered statistically significant.

## Results

The study population consisted of 44 males aged 14–56 years old, with no patients lost to follow-up. All fractures had traumatic causes, with the majority resulting from car accidents. The patients included in the study account for about the 8% of the total scaphoid fractures admitted at the two hospitals during the study. This percentage is higher the 3% registered by other studies [11] but authors believe that the difference between these percentages could be because both hospitals are regional hub for hand surgery and CT scans are always performed in case of scaphoid fractures which could explain the higher percentage diagnosis of complex scaphoid fractures. At one year after surgery, all patients were asymptomatic, and plate removal showed complete fracture consolidation. Three intraoperative complications occurred, all managed during surgery. Flexion and extension range of motion (ROM) significantly improved after plate removal, along with a decrease in QuickDASH scores. It wasn’t possible to evaluate the patients for flexion and extension ROM and QDASH before plate implant due to pain and functional impairment. All patients were instead evaluated for ROM and QDASH before and after four months the plate removal. The flexion ROM before plate removal had a mean value of 62.2° ± 8.8° (range 50°–90°) and improved of 20° at four months reaching a mean value of 83.1° ± 7.2° (range 70°–90°). The extension ROM started at a mean value of 74.2° ± 7.5° (range 50°–80°) showing an improvement of approximately 5° reaching a mean value of 78.8° ± 3.2° (range 70°–80°). As for the QDASH it showed an average improvement of almost 42 points decreasing from a mean score of 54.65 ± 1 2.20 (range 72.73–15.91) to a mean score of 12.69 ± 6.28 (range 6.82–31.82). The improvement of all the three measurement is statistically significant (*P* value < 0.05) according to the paired T tests performed. At four months after plate removal 91% of patients showed an improvement of at least 15° of flexion, 46% of them reached a flexion of 90° and 74% of the total reached a flexion range of at least 80°. Al though 62% of the patients does not show any improvement it must be considered that 56% of the population already had 80° of extension which is the highest possible. Moreover, at four months 88% of patients shows an extension range of 80° and the remaining 12% reached 70° and 75% of patients that did not have an extension range of 80° reached a value of 80° after plate removal. As prove of the goodness of the treatment with volar plating all patients had a QDASH score before plate removal lower than 75 which improved even more at four months after plate removal since 43 out of 44 reached a QDASH score lower than 25 and the only patients not included still improved of almost 30 points going from 61.36 to 31.82. [10, 12].

## Discussion

Comminuted scaphoid fractures can be challenging to diagnose accurately. Before treating a scaphoid fracture it’s always necessary to collect information about the type and the dynamic of trauma (direct injury, high or low energy trauma) related to the age of the patient. Imaging starts with anteroposterior and lateral X-rays of the wrist associated to a specific scaphoid view. CT scans are essential for evaluating the shape and position of scaphoid fragments and the pattern of the fracture because many fractures that look like two-fragment fractures on x-rays actually turn out to be multi-fragment fractures, so a screw could complicate the situation instead of stabilizing the fracture. In that case the angular stability plate is safer and more stable and takes all the fragments.

Surgical intervention offers advantages in achieving precise anatomical alignment and fracture consolidation. Plate and screws system offers biomechanical and biological advantages over other fixation methods, leading to satisfactory functional outcomes comparable to the best outcomes documented in the literature for series treated with screws, surpassing those achieved with staples [4, 6, 8, 9]. We believe that employing plate and screws system offers specific advantages, spanning from biomechanical benefits related to compression at the fracture site, to biological advantages tied to preserving endosteal repair processes, and technical ease in execution.

In the past, the primary indication for plate and screws osteosynthesis unquestionably lay in treating delayed consolidations, avoiding the malunion. In certain cases, in alignment with findings in the literature [13–16], combined treatment with bone grafting may be appropriate when significant bone loss is present in place of graft alone or fixed with screws or staple [17–21].

Only small case series of scaphoid fractures treated with this method have been published in the literature [22–24]. Our case series is the largest in the literature, codifies the indication for plate, and screws osteosynthesis for unstable comminuted recent fractures of the scaphoid body classified as Herbert Type B5 fractures.

Nonetheless, it is worth emphasizing that in scaphoid body fractures, percutaneous synthesis systems lack a strict indication, as they involve completely intra-articular injuries surrounded by hemarthrosis rather than hematoma, and they can be effectively managed with open reduction without impeding the bone healing process. Regarding specifically cannulated screws inserted via a mini retrograde approach, it is worth noting that plate and screws synthesis represents a valid and simpler alternative, requiring significantly less fluoroscopy usage. However, in some cases, screws may lead to scapho-trapezoidal conflict [6].

In a recent study the biomechanical testing shows that plate and screw fixations are equivalent in normal density bone for fixation of a segmental scaphoid defect, locking and angular stability plates are superior to screw fixation in simulated osteoporotic bone models [25, 26].

Some recent studies showed better rotational stability by choosing more than just one screw for scaphoid osteosynthesis [18]. Angular stable plating of scaphoid fractures also provides more rotational stability than single CCS fixation [27].

Even though indications of using screws or plate systems might be different, plate osteosynthesis may be preferable for treatment of dislocated or comminuted fractures or in osteoporotic patients as well as for non-unions [13–16, 25–27].

Volar locking and angular stability plate fixation may offer several advantages over headless screw fixation for scaphoid nonunion, or segmental or comminuted fractures: (1) increased surface area for bony healing; (2) preserved vascularity; and (3) maintenance of a gap for graft insertion. In scaphoid fractures with segmental defect, plate and screw fixation demonstrates similar loads to failure, but plate fixation performs superiorly to screw fixation for gap recovery after an applied load to failure.

In the application of the plate osteosynthesis technique, meticulous capsular reconstruction, along with proper skin suturing, assumes a crucial role. Notably, we did not observe any instances of scar-related pain or cases of algodystrophy. The absence of algodystrophic complications can be attributed, in large part, to the possibility of early mobilization, facilitated by the stability of the synthesis of all the fracture fragments.

According to other studies [24–29], the plate can impinge with the nearby structures, and should be removed once the fracture is consolidated. Although rom and DASH limitation due to impingement was present in all patients it must be specified that it was not symptomatic in all of them. Nevertheless, the authors decided to perform plate removal even in the asymptomatic cases in consideration of the young age of most patients thus avoiding the risk of a secondary fracture with the hardware still implanted. After plate removal, we verified a significant improvement of the range of motion especially in wrist flexion.

## Conclusion

Plate and screws system is a viable and appropriate method of osteosynthesis in the treatment of unstable and comminuted recent fractures occurring in the middle third of the carpal scaphoid.

## Data Availability

No additional data are available.
